# Reply: aromatase inhibition in advanced breast cancer: no conclusive evidence of efficacy differences

**DOI:** 10.1038/sj.bjc.6602435

**Published:** 2005-03-15

**Authors:** R Sainsbury

**Affiliations:** 1 Department of Surgery, Royal Free and University College Medical School, London, UK

**Sir**,

In his letter, Dr Bhatnagar challenges the claim that there were ‘no efficacy differences between letrozole and anastrozole’. Firstly, I would like to point out that this claim was not stated in my review (Sainsbury, 2004). Such a statement on the relative efficacy of two agents could only be assessed by direct comparison in a large randomised, double-blind, clinical trial and no such data are available for letrozole and anastrozole. It is therefore inappropriate to reach conclusions about the relative efficacy of agents on the basis of cross-trial comparisons. The overall claim made in the review was that ‘Together these data suggest that once a certain threshold of aromatase inhibition is reached, small differences in oestrogen suppression between the third-generation aromatase inhibitors (AIs) do not lead to clinically significant differences in overall efficacy’.

This overall claim was based on a review of the best available evidence of clinical efficacy for the third-generation AIs as first- and second-line therapy for advanced breast cancer. The highest level of evidence was provided by an open-label randomised trial comparing letrozole and anastrozole as second-line endocrine therapy in 713 patients with advanced breast cancer (Rose *et al*, 2003). As described in my review, the only end point that showed a significant difference between letrozole and anastrozole was the secondary end point of objective response rate (19.1 *vs* 12.3%, *P*=0.013), although there was no difference in objective response rate (17.3 *vs* 16.8%) for those patients with hormone receptor-positive tumours (48% of the total population and the clinically relevant target population for endocrine therapy). While, as commented by Dr Bhatnagar, a higher likelihood of responding to endocrine therapy is highly relevant for women with advanced breast cancer, this result should not be overinterpreted. This trial was an open-label trial and, therefore, open to bias particularly with regard to subjective end points such as objective response and tolerability. The difference in overall objective response rate did not translate into a benefit for letrozole on the primary end point of time to progression or any other efficacy end point, and is perhaps, therefore, unlikely to result in the delay of other therapies as stated by Dr Bhatnagar. While neither Dr Bhatnagar nor I can explain why the statistically significantly higher objective response rate was limited to the group of patients with undetermined hormone status, the fact remains that in those patients with hormone receptor-positive disease (the clinically relevant group of patients), there was no statistically significant difference in objective response between letrozole and anastrozole.

Dr Bhatnagar states in his letter that in a Phase III study in postmenopausal women receiving first-line therapy for advanced breast cancer, letrozole was found to be superior to tamoxifen in all end points including a prospectively planned survival analysis at 1- and 2-years follow-up. However, in the prospectively planned final analysis of overall survival in this study, at a median follow-up of 32 months, there was no significant difference in overall survival between letrozole and tamoxifen (overall log-rank; *P*=0.53) (Mouridsen *et al*, 2003).

Additional truncated log-rank tests at 6-month intervals did show nominally statistically significant differences in favour of the randomised letrozole arm at 6, 12, 18 and 24 months, but these were nonprotocolled, retrospectively planned analyses (albeit planned before database lock), and were not adjusted for multiple comparisons. As such, any findings should only be considered exploratory as already stated by Dr Buzdar in his comments on this study (Buzdar, 2004). The performance of the ‘gold standard’ tamoxifen treatment in this study has also been questioned (Buzdar, 2002), highlighting again the many difficulties associated with cross-trial comparisons.

Dr Bhatnagar also suggests that letrozole may elicit a response in hormone receptor-negative tumours; however, there appears to be no credible basis for this claim. While benefits with anastrozole are most apparent in the hormone receptor-positive women who receive first-line treatment (Bonneterre *et al*, 2001), its use is indicated in those patients with undetermined hormone receptor status because, as pointed out by Dr Bhatnagar, the majority will be endocrine responsive due to their hormone receptor-positive status. Further, in the overall population, anastrozole is at least equivalent to tamoxifen in terms of median time to progression and is associated with tolerability benefits over tamoxifen (Bonneterre *et al*, 2001). In the second-line Phase III studies, both anastrozole and letrozole were assessed only in patients with hormone receptor-positive or unknown status.

Finally, Dr Bhatnagar queries the hypothesis that although clinical efficacy may be unaffected by small differences in potency, this does not preclude the fact that small differences in oestrogen suppression may lead to differences in side effect profiles. As suggested by Dr Bhatnagar, this statement is purely speculative. A similar statement was made in the ASCO Expert Panel review on adjuvant aromatase inhibitors: ‘…closely related agents with similar mechanisms of action may have different toxicity profiles’ (Winer *et al*, 2002). Dr Bhatnagar continues that the less potent AI anastrozole has been associated with a marked increase in fracture rates when used in early breast cancer (The ATAC Trialists' Group, 2002, 2003), whereas letrozole has not (Goss *et al*, 2003). However, the data from these studies are not comparable. While both studies aimed to compare the safety and efficacy of the AI with tamoxifen in postmenopausal women with early breast cancer, patients in the letrozole trial had already completed 5 years of adjuvant tamoxifen therapy, an agent with a mild protective effect against bone loss due to its partial oestrogen agonist activity (Grey *et al*, 1995) and, furthermore, the comparison was *vs* placebo, not tamoxifen as in the ATAC trial. Nevertheless, the letrozole trial still found a higher incidence of previously undiagnosed osteoporosis and fractures in the letrozole group than the placebo group (Goss *et al*, 2003), and the difference in osteoporosis rates was statistically significant in the updated analysis of this trial presented at ASCO 2004 (8 *vs* 6%, *P*=0.003) (Goss, 2004). At a median follow-up of 37 months, anastrozole was associated with a higher incidence of fractures than tamoxifen in patients who received primary adjuvant therapy for early breast cancer (7.1 *vs* 4.4%, *P*<0.001) (The ATAC Trialists' Group, 2003); however, the fracture rate with anastrozole appears to stabilise after reaching a peak at 2 years and the relative risk *vs* tamoxifen does not worsen with continued treatment (Locker and Eastell, 2003). Anastrozole remains the only AI with data quantifying its effects on bone in this setting.

In summary, the available evidence does suggest that small differences in oestrogen suppression between the third-generation AIs do not lead to clinically significant differences in overall efficacy as concluded in my review (Sainsbury, 2004).

‘The recently presented, but as yet unpublished, results of the Big-Femta (1–98) study demonstrated similar effects on bone for letrozole in the adjuvant setting. There appears to be an, as yet unexplained, excess rate of cardiac events in the letrozole arm’.

Finally, I am challenged to declare my associations with the pharmaceutical industry. Of the endocrine agents I was involved in the clinical trials of goserelin (ZEBRA trial), vorazole and raloxifene as well as the Cancer Research Campaign under 50s trial. I was initially on the International Steering committee (now merged into Steering Committee) of ATAC. I have helped train the UK sales force for Novartis and use all three available aromatase inhibitors in my clinical practice. I have received sponsorship from AstraZeneca, Novartis and Pharmacia (now Pfizer).
